# Supplementary material for: Perceptual Plasticity in Bilinguals: Language Dominance Reshapes Acoustic Cue Weightings
Source: Brain Sci. 2025 Sep 27;15(10):1053. doi: 10.3390/brainsci15101053 (PMC12563407; doi:10.3390/brainsci15101053)
Supplement: Supplementary file 1 [file brainsci-15-01053-s001.zip › brainsci-3799709-supplementary.pdf]

Supplementary Materials

S1. Bilingual Language Profile (Spanish-English, adapted; Birdsong et al., 2012; Gertken et al., 2014)

I. Biographical Information

Participant number (given to you at the beginning of the testing session): ___	Today's Date: _____/_____/_____

Age: _________	☐ Male / ☐ Female	Current place of residence: city/state _______ country______

Highest level of formal education:	☐ Less than high school	☐ High school	☐ Some college
	☐ College (B.A., B.S.)	☐ Some graduate school	☐ Masters
	☐ PhD/MD/JD	☐ Other: _______	

II. Language history

In this section, we would like you to answer some factual questions about your language history by placing a check in the appropriate box.

1. At what age did you **start learning** the following languages?

English

☐ Since birth ☐ 1 ☐ 2 ☐ 3 ☐ 4 ☐ 5 ☐ 6 ☐ 7 ☐ 8 ☐ 9 ☐ 10 ☐ 11 ☐ 12 ☐ 13 ☐ 14 ☐ 15 ☐ 16 ☐ 17 ☐ 18 ☐ 19 ☐ 20+

Spanish

☐ Since birth ☐ 1 ☐ 2 ☐ 3 ☐ 4 ☐ 5 ☐ 6 ☐ 7 ☐ 8 ☐ 9 ☐ 10 ☐ 11 ☐ 12 ☐ 13 ☐ 14 ☐ 15 ☐ 16 ☐ 17 ☐ 18 ☐ 19 ☐ 20+

2. At what age did you **start to feel comfortable** using the following languages?

English

☐ As early as I can remember ☐ 1 ☐ 2 ☐ 3 ☐ 4 ☐ 5 ☐ 6 ☐ 7 ☐ 8 ☐ 9 ☐ 10 ☐ 11 ☐ 12 ☐ 13 ☐ 14 ☐ 15 ☐ 16 ☐ 17 ☐ 18 ☐ 19 ☐ 20+ ☐ Not yet

Spanish

☐ As early as I can remember ☐ 1 ☐ 2 ☐ 3 ☐ 4 ☐ 5 ☐ 6 ☐ 7 ☐ 8 ☐ 9 ☐ 10 ☐ 11 ☐ 12 ☐ 13 ☐ 14 ☐ 15 ☐ 16 ☐ 17 ☐ 18 ☐ 19 ☐ 20+ ☐ Not yet

3. How many years of **classes (grammar, history, math, etc.)** have you had in the following languages (primary school through university)?

English

☐ 0 ☐ 1 ☐ 2 ☐ 3 ☐ 4 ☐ 5 ☐ 6 ☐ 7 ☐ 8 ☐ 9 ☐ 10 ☐ 11 ☐ 12 ☐ 13 ☐ 14 ☐ 15 ☐ 16 ☐ 17 ☐ 18 ☐ 19 ☐ 20+

Spanish

☐ 0 ☐ 1 ☐ 2 ☐ 3 ☐ 4 ☐ 5 ☐ 6 ☐ 7 ☐ 8 ☐ 9 ☐ 10 ☐ 11 ☐ 12 ☐ 13 ☐ 14 ☐ 15 ☐ 16 ☐ 17 ☐ 18 ☐ 19 ☐ 20+

4. Have you ever lived in **Mexico**? If your answer is *yes*, how many years did you spend / have you spent living there?

- ☐ Yes (time spent living in Mexico (in years): _________)
- ☐ No, I have never lived in Mexico

5. Have you ever lived in **The United States**? If your answer is *yes*, how many years did you spend / have you spent living there?

- ☐ Yes (time spent living in the US (in years): _________)
- ☐ No, I have never lived in the US

6. Have you ever been a **border commuter** (i.e. someone who lives in Mexico, but studies/works in The United States (or vice versa), and commutes to school/work at least a couple times a week?) If your answer is *yes*, how many years have you spent as a border commuter?

- ☐ Yes (time spent as a border commuter (in years): ________)
- ☐ No, I have never been a border commuter

7. How many years have you spent living in a **family** where its members (parents, siblings, etc.) are bilingual in Spanish and English?

☐	☐	☐	☐	☐	☐	☐	☐	☐	☐	☐	☐	☐	☐	☐	☐	☐	☐	☐	☐	☐
0	1	2	3	4	5	6	7	8	9	10	11	12	13	14	15	16	17	18	19	20+

8. How many years have you spent in a **school and/or work environment** where it is common to speak English and Spanish in a same day?

0	1	2	3	4	5	6	7	8	9	10	☐	☐	☐	☐	☐	☐	☐	☐	☐	☐
											11	12	13	14	15	16	17	18	19	20+

III. Language use

In this section, we would like you to answer some questions about your language use by placing a ***circle*** *around the appropriate box. Total use for all languages in a given question should equal 100%. For example:*

English	0%	10%	20%	30%	40%	50%	60%	**70%**	80%	90%	100%
Spanish	100%	90%	80%	70%	60%	50%	40%	**30%**	20%	10%	0%

9. In an average week, what percentage of the time do you use the following languages **with friends**?

English	0%	10%	20%	30%	40%	50%	60%	70%	80%	90%	100%
Spanish	100%	90%	80%	70%	60%	50%	40%	30%	20%	10%	0%

10. In an average week, what percentage of the time do you use the following languages **with family**?

English	0%	10%	20%	30%	40%	50%	60%	70%	80%	90%	100%
Spanish	100%	90%	80%	70%	60%	50%	40%	30%	20%	10%	0%

11. In an average week, what percentage of the time do you use the following languages **at school/work**?

English	0%	10%	20%	30%	40%	50%	60%	70%	80%	90%	100%
Spanish	100%	90%	80%	70%	60%	50%	40%	30%	20%	10%	0%

12. When you talk to yourself, how often do you **talk to yourself** in the following languages?

English	0%	10%	20%	30%	40%	50%	60%	70%	80%	90%	100%
Spanish	100%	90%	80%	70%	60%	50%	40%	30%	20%	10%	0%

13. When you count, how often do you **count** in the following languages?

English	0%	10%	20%	30%	40%	50%	60%	70%	80%	90%	100%
Spanish	100%	90%	80%	70%	60%	50%	40%	30%	20%	10%	0%

IV. Language proficiency

In this section, we would like you to rate your language proficiency by giving marks from 0 to 6.

0= not well at all *6= very well*

14. a. How well do you speak **English**? ☐ 0 ☐ 1 ☐ 2 ☐ 3 ☐ 4 ☐ 5 ☐ 6

b. How well do you speak **Spanish**? ☐ 0 ☐ 1 ☐ 2 ☐ 3 ☐ 4 ☐ 5 ☐ 6

15. a. How well do you understand **English**? ☐ 0 ☐ 1 ☐ 2 ☐ 3 ☐ 4 ☐ 5 ☐ 6

b. How well do you understand **Spanish**? ☐ 0 ☐ 1 ☐ 2 ☐ 3 ☐ 4 ☐ 5 ☐ 6

16. a. How well do you read **English**? ☐ 0 ☐ 1 ☐ 2 ☐ 3 ☐ 4 ☐ 5 ☐ 6

b. How well do you read **Spanish**? ☐ 0 ☐ 1 ☐ 2 ☐ 3 ☐ 4 ☐ 5 ☐ 6

17. a. How well do you write **English**? ☐ 0 ☐ 1 ☐ 2 ☐ 3 ☐ 4 ☐ 5 ☐ 6

b. How well do you write **Spanish**? ☐ 0 ☐ 1 ☐ 2 ☐ 3 ☐ 4 ☐ 5 ☐ 6

V. Language attitudes

In this section, we would like you to respond to statements about language attitudes by giving marks from 0-6.

0=disagree *6=agree*

18. a. I feel like myself when I speak **English.** ☐ 0 ☐ 1 ☐ 2 ☐ 3 ☐ 4 ☐ 5 ☐ 6

b. I feel like myself when I speak **Spanish.** ☐ 0 ☐ 1 ☐ 2 ☐ 3 ☐ 4 ☐ 5 ☐ 6

19. a. I identify with an **English-speaking** culture. ☐ 0 ☐ 1 ☐ 2 ☐ 3 ☐ 4 ☐ 5 ☐ 6

b. I identify with a **Spanish-speaking** culture. ☐ 0 ☐ 1 ☐ 2 ☐ 3 ☐ 4 ☐ 5 ☐ 6

20. a. It is important to me to use (or eventually use) **English** like a native speaker. ☐ 0 ☐ 1 ☐ 2 ☐ 3 ☐ 4 ☐ 5 ☐ 6

b. It is important to me to use (or eventually use) **Spanish** like a native speaker. ☐ 0 ☐ 1 ☐ 2 ☐ 3 ☐ 4 ☐ 5 ☐ 6

21. a. I want others to think I am a native speaker of **English**. ☐ 0 ☐ 1 ☐ 2 ☐ 3 ☐ 4 ☐ 5 ☐ 6

b. I want others to think I am a native speaker of **Spanish**. ☐ 0 ☐ 1 ☐ 2 ☐ 3 ☐ 4 ☐ 5 ☐ 6

S2. English Proficiency Test: Cloze Test (Brown, 1980)

Test

In the following text, some of the words have been replaced by blanks numbered 1 through 50. First, read the complete text in order to understand it. Then reread it and choose the correct word to fill each blank from the answer sheet. Mark your answers by circling your choice on the answer sheet, not by filling in the blanks in the text.

Man and His Progress

Man is the only living creature that can make and use tools. He is the most teachable of living beings, earning the name of Homo sapiens. (1) __________ ever restless brain has used the (2) __________ and the wisdom of his ancestors (3) __________ improve his way of life. Since (4) __________ is able to walk and run (5) __________ his feet, his hands have always (6) __________ free to carry and to use (7) __________. Man's hands have served him well (8) __________ his life on earth. His development, (9) __________ can be divided into three major (10) __________, is marked by several different ways (11) __________ life.

Up to 10,000 years ago, (12) __________ human beings lived by hunting and (13) __________. They also picked berries and fruits, (14) __________ dug for various edible roots. Most (15) __________, the men were the hunters, and (16) __________ women acted as food gatherers. Since (17) __________ women were busy with the children, (18) __________ men handled the tools.

In a (19) __________ hand, a dead branch became a (20) __________ to knock down fruit or to (21) __________ for tasty roots. Sometimes, an animal (22) __________ served as a club, and a (23) __________ piece of stone, fitting comfortably into (24) __________ hand, could be used to break (25) __________ or to throw at an animal. (26) __________ stone was chipped against another until (27) __________ had a sharp edge. The primitive (28) __________ who first thought of putting a (29) __________ stone at the end of a (30) __________ made a brilliant discovery: he (31) __________ joined two things to make a (32) __________ useful tool, the spear. Flint, found (33) __________ many rocks, became a common cutting (34) __________ in the Paleolithic period of man's (35) __________. Since no wood or bone tools (36) __________ survived, we know of this man (37) __________ his stone implements, with which he (38) __________ kill animals, cut up the meat, (39) __________ scrape the skins, as well as (40) __________ pictures on the walls of the (41) __________ where he lived during the winter.

(42) __________ the warmer seasons, man wandered on (43) __________ steppes of Europe without a fixed (44) __________, always foraging for food. Perhaps the (45) __________ carried nuts and berries in shells (46) __________ skins or even in light, woven (47) __________. Wherever they camped, the primitive people (48) __________ fires by striking flint for sparks (49) __________ using dried seeds, moss, and rotten (50) __________ for tinder. With fires that he kindled himself, man could keep wild animals away and could cook those that he killed, as well as provide warmth and light for himself.

Answers

JD's cloze test "Man and his progress" - answer keys		
	Exact	***Acceptable***
1	his	man's, our, the
2	knowledge	accomplishments, culture, cunning, examples, experience(s), hands, ideas, information, ingenuity, instinct, intelligence, mistakes, nature, power, skill(s), talent, teaching, technique, thought, will, wit, words, work
3	to	
4	man	he
5	on	upon, using, with
6	been	felt, hung, remained
7	tools	adequately, carefully, conventionally, creatively, diligently, efficiently, freely, implements, objects, productively, readily, them, things, weapons
8	during	all, for, improving, in, through, throughout, with
9	which	also, basically, conveniently, easily, historically, however, often, since, that, thus
10	periods	areas, categories, divisions, eras, facets, groups, parts, phases, sections, stages, steps, topics, trends
11	of	for, in, through, towards
12	all	early, hungry, many, most, only, primitive, the, these
13	fishing	farming, foraging, gathering, killing, scavenging, scrounging, sleeping, trapping
14	and	often, ravenously, some, the
15	often	always, emphatically, important, nights, normally, of, times, trips
16	the	all, house, many, most, older, their, younger
17	the	all, many, married, most, often, older, primate, these
18	the	all, constructive, many, most, older, primate, tough, younger
19	man's	able, big, closed, coordinated, creative, deft, empty, free, human('s), hunter's, learned, needed, needy, person's, right, single, skilled, skillful, small, strong, trained
20	tool	club, device, instrument, pole, rod, spear, stick, weapon
21	dig	burrow, excavate, probe, search, test
22	bone	arm, easily, foot, had, hide, horn, leg, skull, tail, tusk
23	sharp	big, chipped, fashioned, flat, hard, heavy, large, rough, round, shaped, sizeable, small, smooth, solid, strong, soft, thin,
24	the	a, his, man's, one('s)
25	nuts	apart, bark, bones, branches, coconuts, down, firewood, food, heads, ice, items, meat, objects, open, rocks, shells, sticks, stone, things, tinder, trees, wood
26	one	a, each, flat, flint, glass, hard, obsidian, shale, softer, some, the, then, this
27	it	each, one, they
28	man	being, creature, human's, hunter, men, owner, people, person
29	sharp	glass, hard, jagged, large, lime, pointed, sharpened, small
30	stick	bone, branch, club, log, pole, rod, shaft

31	had	accidentally, cleverly, clumsily, conveniently, creatively, dexterously, double, easily, first, ingeniously, securely, simply, soon, suddenly, tastefully, tightly, then, would
32	very	bad, extremely, good, hunter's, incredibly, intelligent, long, modern, most, necessarily, new, portentously, quite, tremendously, useful
33	in	all, among, amongst, by, inside, on, that, using, within
34	tool	device, edge, implement, instrument, item, material, method, object, piece, practice, stone, utensil,
35	development	age, ancestry, discoveries, era, evolution, existence, exploration, history, life, time
36	have	actually, apparently, ever
37	by	and, for, from, had, made, through, used, using
38	could	did, would
39	and	carefully, help, or, skillfully, then, would
40	draw	carve, create, drawing, engrave, hang, paint, painting, place, sketch, some, the
41	cave(s)	animals, place(s), room
42	in	and, during, with
43	the	across, aimless, all, barren, in, dry, flat, high, long, many, plain, stone, through, to, toward, unknown, various,
44	home	appetite, camp, course, destination, destiny, diet, direction, domain, foundation, habitat, income, knowledge, location, lunch, map, meal, path, pattern, place, plan, route, supplement, supply, time, weapons,
45	women	children, families, group, human, hunter, man, men, people, primitives, voyager, wanderers, woman,
46	or	and, animal, animal's, covered, in, like, of, on, their, using, with
47	baskets	bags, blankets, chests, cloth(es), fabric, garments, hides, material, nets, pouches, sacks
48	made	began, built, lighted, lit, produced, started, used
49	and	also, by, occasionally, or, then, together, while
50	wood	bark, branches, dung, forage, grass, leaves, lumber, roots, skin, timber, tree(s)

S3. Relationship between Bilingual Language Profile and Proficiency

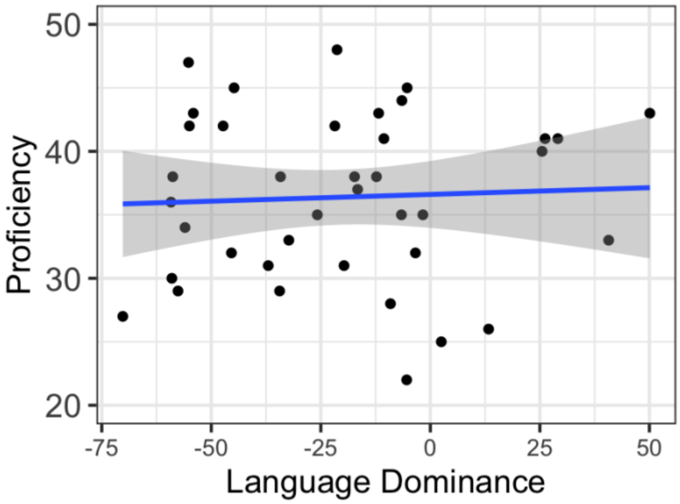

Figure S1. Relationship between language dominance scores and cloze test scores

S4. Spanish Listeners' Individual Responses on the Cue-Weighting Task

Vowel Quality by Pitch

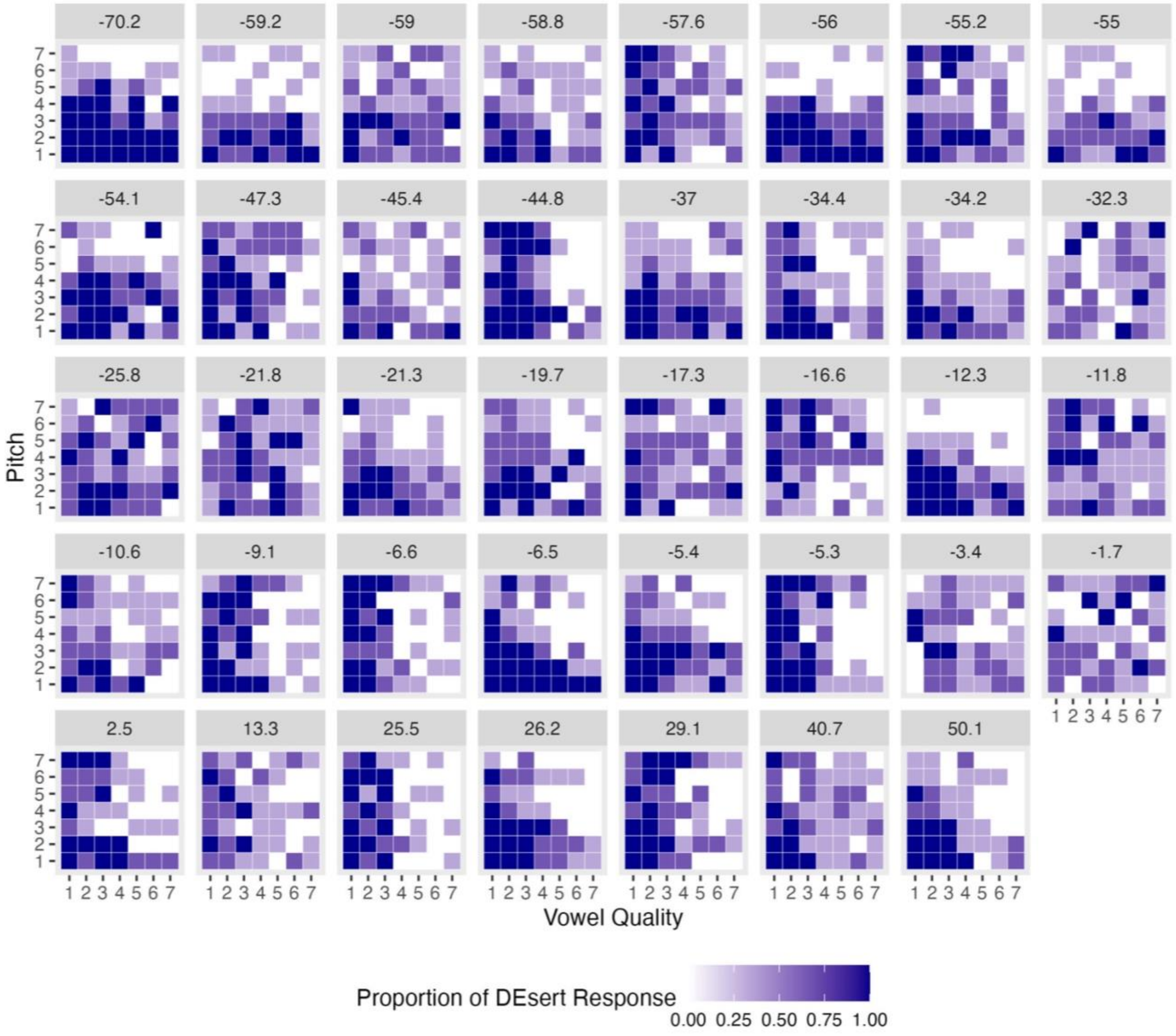

Figure S2. Individual bilingual listeners' proportions of *DEsert* selection when the stimuli varied by vowel quality and pitch; participants are labeled with their language dominance scores.

Vowel Quality by Duration

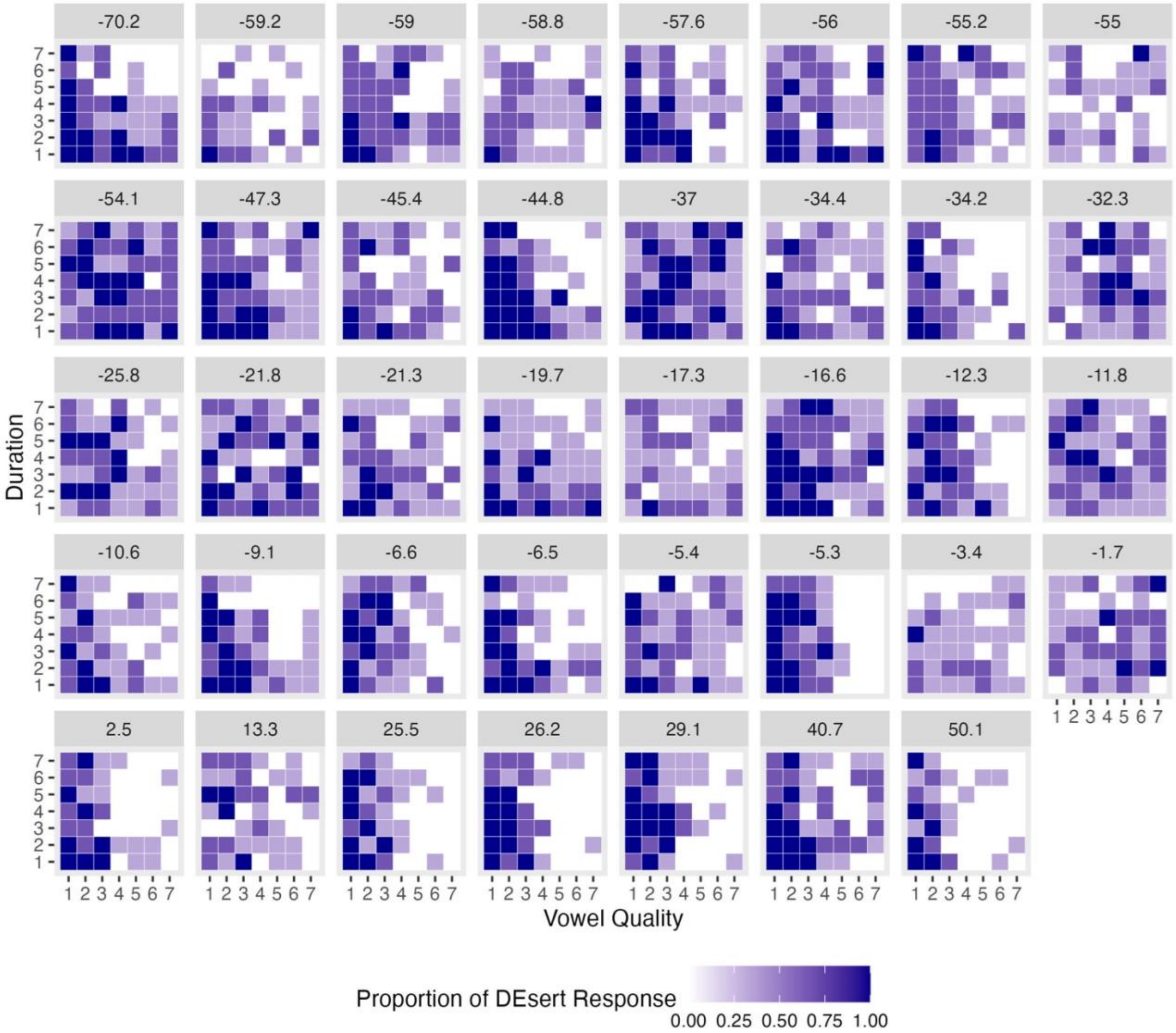

Figure S3. Individual bilingual listeners' proportions of *DEsert* selection when the stimuli varied by vowel quality and duration; participants are labeled with their language dominance scores.

Pitch by Duration

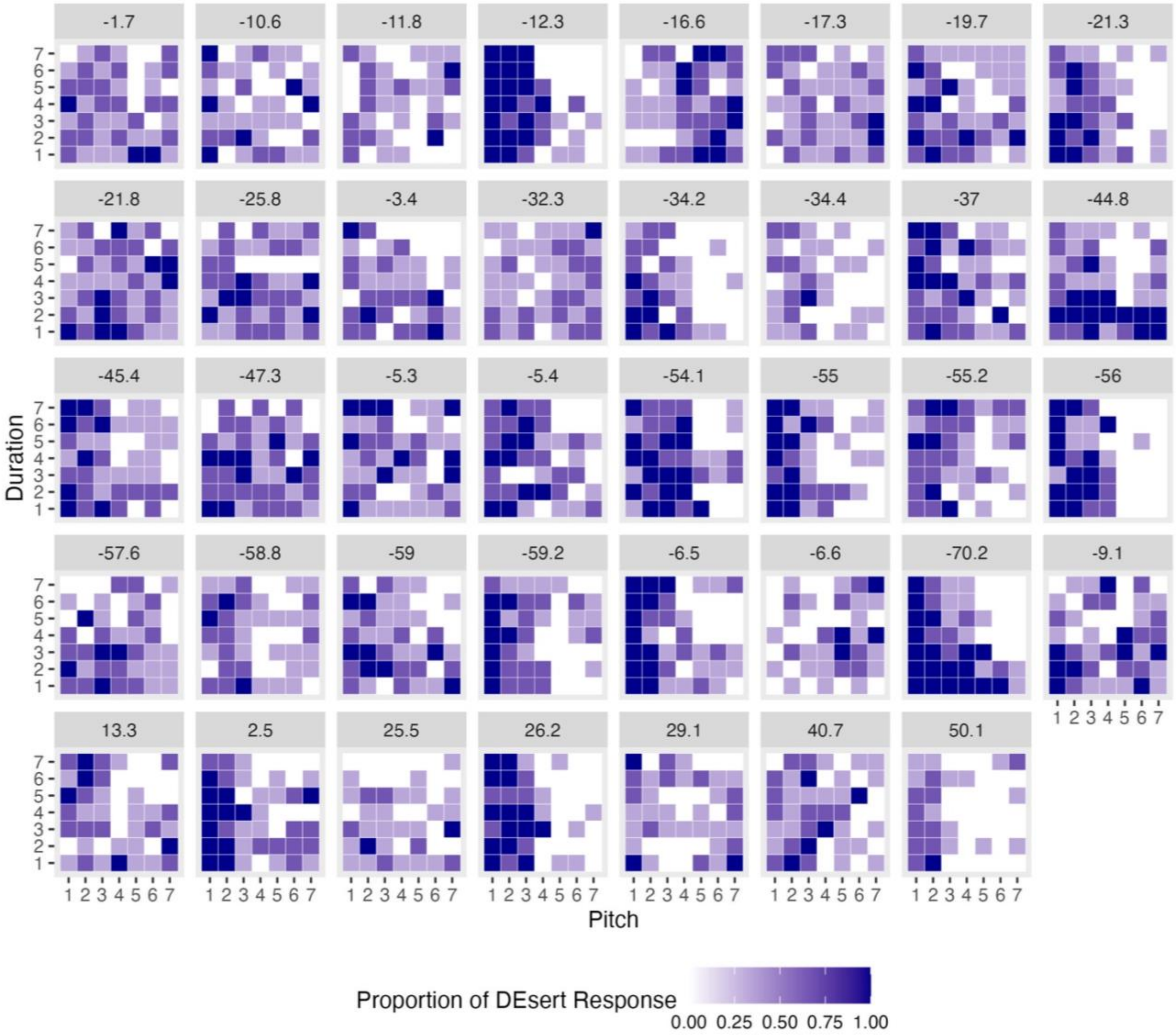

Figure S4. Individual bilingual listeners' proportions of *DEsert* selection when the stimuli varied by pitch and duration; participants are labeled with their language dominance scores.

S5. Bayesian Models on Bilinguals' Responses with Language Dominance as Fixed Effect

Vowel Quality by Pitch Stimuli

Table S1. Results of Bayesian Logistic Regression on Bilinguals' Responses When Stimuli Varied by Vowel Quality and Pitch

Parameter	Posterior Mean	95% CI (Lower)	95% CI (Higher)	$P(\beta > 0)$	$P(\beta < 0)$
Intercept	–0.058	–0.186	0.067	.177	.823
**Vowel Quality**	–0.367	–0.419	–0.317	<.001	**>.999**
**Pitch**	–0.277	–0.328	–0.226	<.001	**>.999**
Language Dominance	–0.064	–0.159	0.031	.090	0.909
Vowel Quality × Pitch	0.015	–0.011	0.041	.869	0.131
**Vowel Quality × Language Dominance**	–0.114	–0.146	–0.083	<.001	**>.999**
**Pitch × Language Dominance**	0.080	0.050	0.111	**>.999**	<.001
Vowel Quality × Pitch × Language Dominance	0.002	–0.014	0.018	.613	0.295

Note. Bolded fixed effects are credible.

Vowel Quality by Duration Stimuli

Table S2. Results of Bayesian Logistic Regression on Bilinguals' Responses When Stimuli Varied by Vowel Quality and Duration

Parameter	Posterior Mean	95% CI (Lower)	95% CI (Higher)	$P(\beta > 0)$	$P(\beta < 0)$
Intercept	–0.243	–0.410	–0.077	.002	.998
**Vowel Quality**	–0.390	–0.432	–0.348	<.001	**>.999**
**Duration**	–0.164	–0.206	–0.123	<.001	**>.999**
**Language Dominance**	–0.149	–0.307	0.005	.028	**.972**
**Vowel Quality × Duration**	0.026	0.004	0.047	**.990**	.010
**Vowel Quality × Language Dominance**	–0.104	–0.136	–0.072	<.001	**>.999**
**Duration × Language Dominance**	0.032	0.001	0.062	**.979**	.021
Vowel Quality × Duration × Language Dominance	0.005	–0.011	0.021	.750	.250

Note. Bolded fixed effects are credible.

Pitch by Duration Stimuli

Table S3. Results of Bayesian Logistic Regression on Bilinguals' Responses When Stimuli Varied by Pitch and Duration

Parameter	Posterior Mean	95% CI (Lower)	95% CI (Higher)	$P(\beta > 0)$	$P(\beta < 0)$
Intercept	–0.287	–0.426	–0.153	<.001	>.999
**Pitch**	–0.285	–0.330	–0.238	<.001	**>.999**
**Duration**	–0.125	–0.171	–0.079	<.001	**>.999**
**Language Dominance**	–0.158	–0.275	–0.043	0.005	**0.995**
Pitch × Duration	–0.004	–0.027	0.019	0.350	0.650
**Pitch × Language Dominance**	0.060	0.030	0.089	**>.999**	<.001
**Duration × Language Dominance**	0.025	–0.003	0.054	**0.959**	0.041
Pitch × Duration × Language Dominance	–0.006	–0.020	0.008	0.208	0.792

Note. Bolded fixed effects are credible.
